# How Can We Create Osler’s “Great Physician”? Fundamentals for Physicians’ Competency in the Twenty-first Century

**DOI:** 10.1007/s40670-020-01003-1

**Published:** 2020-06-15

**Authors:** Gabriel M. Ronen, Olaf Kraus de Camargo, Peter L. Rosenbaum

**Affiliations:** 1 Faculty of Health Sciences, Department of Pediatrics, McMaster University, HSC Rm. 3A58, 1200 Main Street West, Hamilton, Ontario L8S 4K1 Canada; 2 CanChild Centre for Childhood Disability Research, Hamilton, Canada; 3 Ron Joyce Children’s Health Centre, Hamilton, Canada

Providing excellent clinical care, as Sir William Osler would have valued, is ultimately about understanding and addressing effectively the human dramas of personal predicaments (including those experienced by healthcare providers) [1]. In this essay, we argue that, without sacrificing the teaching of any of the best of current biomedical science to physicians-in-training, there is an urgent need for integrated, complementary training in three parallel themes. These themes should address international concepts of health and functioning (WHO’s ICF) [2]; an understanding of the individual human experience of illness and how to help patients report outcomes meaningful to them (PROMs) [3]; and a recognition of the ethical dimensions of everyday clinical practice [4]. These concepts are recognized as important in Canada’s CanMeds description of key competencies for physicians, as illustrated briefly below [5].

In order to teach and to learn these concepts, we need to fuse medical sciences with practical frameworks that actively incorporate and *integrate* the humanities into medicine. Teaching learners both to recognize and to become comfortable with these aspects of medicine is essential, and there are multiple opportunities to do this well. We argue that if these concepts are introduced early in education and training, reinforced as granular elements of all professional training, and modeled for learners by health professional leaders, there will be a powerful transformation of the whole health system, led by a new generation of enlightened and open-minded professionals. The widespread integration into medical training of the ICF, specific attention to patients’ voices, and everyday ethics not only poses significant challenges, but equally importantly represents a substantial opportunity [6].

## Introduction of the Issue

One usually chooses to become a health professional out of the desire to interact with and help “sick” people. Yet medical curricula are, to an ever-increasing extent, guided by the rapid discovery of a multitude of “-omics.” Modern-day teaching puts a premium on biomedical sciences and conveys to learners the impression that so-called personalized (precision) medicine can be achieved if one only gathers enough biomedical details [7]. Contemporary medical education focuses extensively on “science” and “research evidence,” often associated with reductionist thinking, and pays significantly less formal attention to training learners about the human predicaments (including suffering) experienced by people with health concerns. Thus, for example, medical schools are primarily rated by their research output (https://www.usnews.com/education/best-graduate-schools/articles/medical-schools-methodology).

Medical educators often cite Sir William Osler to encourage healthcare students and professionals to practice medicine in a humane [8], holistic, and individualized manner—the presumed goal of medical schools. Osler is credited with saying that “The good physician treats the disease; the great physician treats the patient who has the disease.” In his essay “The tyranny of the idea of cure,” Ronnie Mac Keith cautioned that “Patients are not uninterested vehicles of interesting diseases” [9]. The “greatness” proposed by Osler and emphasized by Mac Keith can, we believe, be achieved by a shift of focus from “disease” to the “individual patient.”

George L Engel is widely recognized for conceptualizing the idea of “biopsychosocial health” frequently endorsed in medical curricula as embracing the model of health promoted by Osler [10]. Today one can replace many components of the ill-defined label of “the art of medicine” [11, p. 1188] by well-researched, well-established, evidence-based approaches to clinical care that will enhance the goal of achieving humane and holistic aspects of the practice of medicine. In his moving reflection “The nature of suffering and the goals of medicine” (1982), Eric Cassel identified another component necessary to make the “great physician” [12]. He posited that suffering is a spiritual or existential experience of the “person,” separate from corporeal issues such as pain or discomfort, and argued that clinical medicine often fails both to recognize and to address “suffering.” He identified distinctions between pain and suffering, and proposed ways for clinicians to become attuned to this human experience: how to identify it, and how to support each unique individual through their medical care services by addressing their suffering.

As far back as Hippocrates (460–370 BC), physicians were admonished to “Cure sometimes, treat often, comfort always.” Indeed, when patients are asked what they value in a physician encounter, they “first and foremost … are thankful for their providers’ medical care. Second, and very closely related, they are thankful for personality and demeanour” [13].

**Fig. 1:**
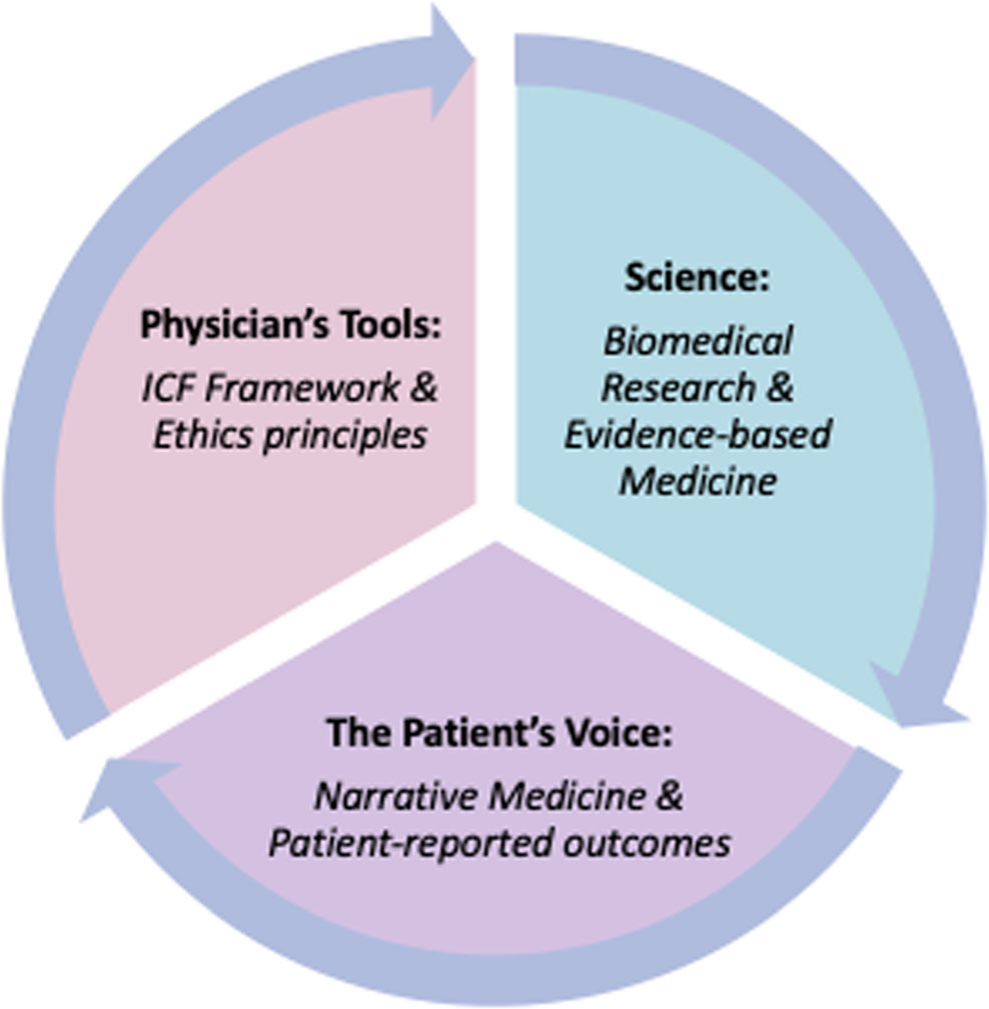
A suggested model for “*Osler’s* Great Physician” framework

Gordon [14] asked the fundamental question: “What kind of curriculum could offer the best preparation for times of psychological and moral duress? Biomedicine falls silent. What might the humanities offer?” In this essay, we propose modest answers to Gordon, involving three interrelated approaches to the human side of clinical care. These can be woven into the fabric of medical education to inform and infuse the training of all health professionals from the start of their careers. These ideas are argued to be complementary—orthogonal—to standard curricular content for any health profession (Fig. 1).

## The Challenges

**Fig. 2:**
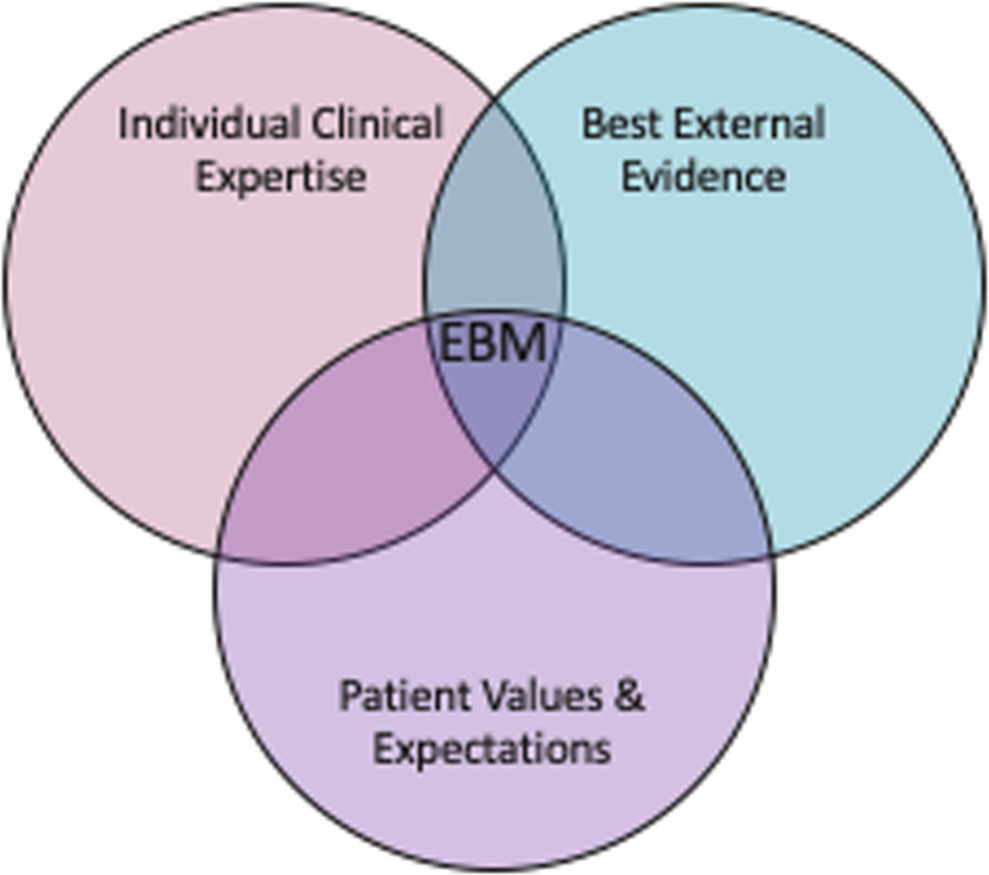
Model of evidence-based medicine

Recognizing the limited scientific evidence of many aspects of medical practice, Sackett et al. [15] argued that evidence-based practice is not a “cookbook” for medicine but needs to consider and include the clinical expertise and judgment of the physician and *the values expressed by the patient* (emphasis from the authors) (Fig. 2) [16]. Initiatives to improve the performance of physician trainees in their interactions with patients may include the use of “patient-actors” in structured assessments; simulations; and an emphasis on moving from a paternalistic to a more collaborative model of care [17].

In modern curricula, trainees need both to demonstrate good clinical examination, reasoning, and problem-solving skills and to be able to interact in a professional and humane way with patients. Despite this awareness, the educational and training content related to empathy, humanity, tenderness, communication skills, shared decision-making, and related social sciences is usually taught in separate, so-called professional competencies units. These are often disembodied modules not fully integrated into the clinical-biological content. However, to develop excellence in these clinical skills, it is fundamentally important for learners to acquire a deeper understanding of their patients’ lives [18]. Ongoing clinical experience often allows people to develop such an understanding over years of practice, but we believe that training for all of today’s health professionals requires frameworks and tools that equip newly minted physicians with the ability to appraise biomedical science critically, collect holistic information about their patients systematically, and engage with patients ethically in shared decision-making processes.

Another challenge is that conducting studies into the effects of any curricular innovation is complicated by a number of overwhelming methodological hindrances [19]. In Ousager and Johannessen’s 2010 systematic review of 245 articles concerning the humanities in medical education, only nine papers provided evidence of attempts to document their long-term impact. The authors concluded that this sparse documentation may threaten the continued development in humanities-related progress in medical education in today’s climate that demands evidence to demonstrate educational effectiveness [20].

In the next section, we describe three well-established principles the authors have successfully taught and practiced that can help to equip professionals to acquire, systematically, the competencies to become “great physicians.”

## Key Contemporary Concepts: Opportunities for Application in Medical Education

### How Can the WHO’s Concepts About Health, as Embodied in the Framework of the ICF [2], Support a Student to Become a “Great Physician”?

**Fig. 3:**
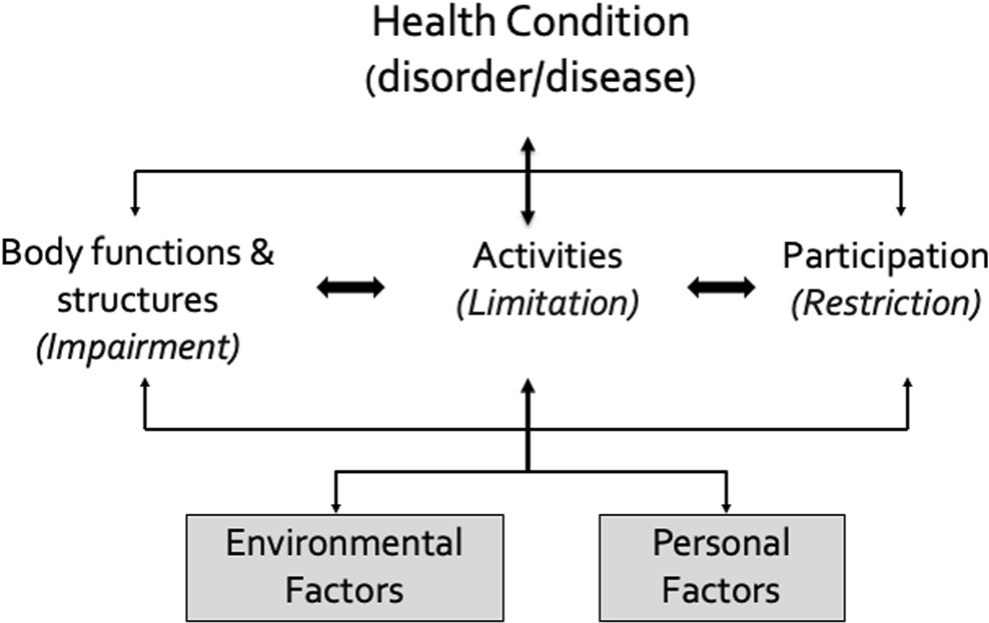
The ICF framework [2]

The ICF provides a structure for the concept of biopsychosocial health in a concise, practical, and comprehensive framework. It has been heralded as an important step toward a shared understanding of functioning and disability, integrating transdisciplinary concepts and terminology into a transcultural, universal description of health. The framework illustrates both the integration of and interactions among the elements of the biomedical model of health (*body functions and structures*) and those of the social model of health: *activities* (tasks and demands of daily life), and *participation* (meaningful engagement in life situations). These components of functioning exist in a dynamic relationship with each other as influenced by the ever-present individual contextual factors of the physical and social *environment(s)*, including family and peer support, and *personal factors* such as self-efficacy and resilience (Fig. 3) [2].

The ICF attributes include:

(A) An internationally crafted practical, non-categorical universal approach to *health and functioning*;
(B) An all-encompassing framework to describe “disease and person-in-context,” including an integrated biopsychosocial framework that allows practitioners to identify and “rule in” elements of person and environment; these then create an inclusive and useful understanding of the health condition in the individual experiencing it, including the personal and contextual realities of that person (i.e., complementary to the implications of the condition as viewed biomedically);
(C) Facilitation of the identification and organization of issues, problems, and strengths into a coherent “whole”;
(D) Multiple points of entry for interventions—beyond the traditional biomedical level; and
(E) A way of thinking that has increasing uptake by healthcare professionals in many areas of medicine, including interprofessional education and collaboration for both clinical care and health services research [21].

The ICF is framed as a classification that applies to all individuals, rather than exclusively to people experiencing disability resulting from a biomedical event [22].

**Example**: in Canada, the CanMeds Health Advocate Key Competencies #1 and #2 expect clinicians to “Respond to an individual patient’s health needs by advocating with the patient within and beyond the clinical environment” and to “Respond to the needs of the communities or populations they serve by advocating with them for system-level change in a socially accountable manner.” Applying the ICF in clinical practice enables patients to identify their needs, describe particular barriers to participation, and identify potential facilitators that might lie beyond impairments of body functions and structures as usually assessed by healthcare providers (in effect, making explicit any “social determinants of health” in the individual situation). The ICF framework thus guides the clinician systematically toward a holistic appraisal of the person, their predicament, and relevant circumstances to be considered [23].

**Example**: one approach we have explored to teach the application of the ICF is to assign students a “standard” paper problem for their clinical analysis (for 30 min, for example); then introduce the ICF framework and concepts; and then have the students revisit the same problem and undertake an ICF-based case analysis.

***Anticipated outcome: Physicians familiar with the ICF will learn how to think holistically, and to “rule in” relevant aspects of a patient’s reality, and will become good health advocates.***

### How Do we Elicit the Voices of People?

Patient engagement in healthcare and healthcare research is increasingly valued and expected.

**Example**: Medical Expert Key Competency #2 in Canada’s CanMeds 2015: *“*Perform a patient-centred clinical assessment and establish a management plan” [5]. When identifying issues and assessing the impact of interventions, there is a major shift to move beyond prioritizing the perspectives and values of healthcare professionals. Narrative medicine and *patient-reported outcomes* (PROs) emphasize the importance of each patient’s personal perspectives when coming to decisions about potential interventions and management of their issues, and what outcomes will be important to them.

We believe that the skills needed to be able to understand the voice of the person behind the health issue can be taught with the use of trained simulated patients.

**Example**: simulated patients can receive scripts that include examples of valued activities and social participation and then be interviewed by students and provide feedback about the extent to which students have been able to gain insights into them as people, and offer advice about how to improve this dimension of their clinical skills.

#### Narrative Medicine

Listening carefully to patients’ stories not only enriches the knowledge of their physical and psychological condition but also allows healthcare professionals to gain information with which to formulate the diagnosis and propose a management plan. “With narrative competence, physicians can reach and join their patients in illness, recognize their own personal journeys through medicine (and life), acknowledge kinship with and duties toward other healthcare professionals, and inaugurate consequential discourse with the public about health care” [24].

***Anticipated outcome: The “great physician” seeks out, and values, the patient’s voice.***

#### Patient-reported outcomes (PROs)

PROs embrace any aspect of a personal report of the individual’s health that comes directly, unfiltered, from that person [3]. These reports explicitly reflect the person’s life experience and values, in relation to their health condition and its management, without interpretation of these responses by healthcare professionals or others. Today, PROs are considered the criterion standard to evaluate patients’ outcomes, and they play an essential role in person- and family-centered healthcare [25]. It is equally important to identify the content of structured patient-reported *measures* (*PROMs*) used to assess outcomes of interest. PROMs explicitly refer to standardized and validated person- (patient-) reported outcome measures (including any self-reported scales or items) that cannot be directly captured through other means.

By using PROMs, healthcare providers can focus on a person’s individual health goals and guide diagnostic and management decisions (an illustration of an additional humanistic perspective on “personalized medicine”). Through PROMs, healthcare providers are able to learn whether patients share our professional perspectives about their health and wellbeing, or whether they have additional or different concerns, and what those are. PROMs can teach us whether, in their own eyes, our patients are truly better after interventions, by following the ethical principle of doing more good than harm within the context of their own lives. Healthcare providers would be more likely to use PROMs routinely if these were seen, during medical school training, to be applied in the context of each patient scenario.

***Anticipated outcome: Physicians familiar with PROMs learn how to value their patients’ voices and will be good medical experts.***

### Are We Aware of—Do We Teach—Ethical Perspectives, and Do We Model Them in Everyday Practice?

Ethics occupies a central role in all clinical decision-making and is therefore critical to the professional development of the physician. Nonetheless, “ethics” as a topic in health education and healthcare is often thought of in the context of research studies (e.g., the need for “ethics approval”), or of headline-grabbing predicaments that are perceived to require court adjudication for the resolution of complex clinical dilemmas. What is also required—and unfortunately rarely taught—are the ethical concepts and threads that run through all of everyday clinical care [4]. Ethical principles assist in organizing reflections on the moral issues that often arise in clinical practice: examples include decisions about investigations, therapies, intensity of treatment, and whether and how to incorporate patients’ voices. Here the line between good clinical practice and ethics is often blurred—in part because they are so intertwined [4]. Active ethical questioning and deliberation are an essential part of good practice, and hence should be an active ingredient in medical education. CanMeds Professional Key Competency # 1 expects physicians to “Demonstrate a commitment to patients by applying best practices and adhering to high ethical standards.” These concepts should include the clinician’s knowledge and skills, partnership with families and professional team members, communication, honesty, humility, and trust.

Ethical concepts are generally discussed along the lines articulated by Beauchamp and Childress [26], whose four principles include the need to consider beneficence (doing good), non-maleficence (not doing harm), autonomy (independence of the person), and justice (fairness).

**Examples**: practice how to communicate uncertainties or convey difficult news in varied clinical scenarios. Like the ICF concepts described above, we strongly believe that these cross-cutting and over-arching ideas should become part of the fabric of all health professional education for practice—a lens through which to refract all clinical thinking and especially decision-making. An approach to introducing these ideas and engaging learners could include providing a standard clinical “predicament” and challenging the learners to “parse” the story for its “ethical dimensions.” If this were done “cold” as a “takeaway” exercise, students would need to discover and apply principles of ethical thinking. One could then assess the carry-over and generalization of this learning to other situations (such as, for example, a second case example a month later).

***Anticipated outcome: Physicians familiar with ethical principles are well equipped to engage in shared decision-making and will be professionals who are in tune with contemporary social standards.***

## Conclusions

To learn and practice the concepts promoted in this essay, trainees could be encouraged to discuss and analyze, in every clinical scenario, the biopsychosocial components of the ICF as they apply to this specific story; to identify relevant potential interventions; to explore whether the clinician outcomes of interest match those the patient might envision and propose, based on an exploration of the patient’s own views; and finally, to identify the ethical dimensions and possible tensions arising from each decision node of the scenario and how one might approach these. We strongly believe that helping students acquire a mindset to do these exercises will serve them well for the entire careers as these approaches become second nature. We recognize that many faculty may understand these ideas but be unsure how best to teach, promote, and model them. Integrating these ideas into medical school curricula could thus become the focus of a journey shared by both new and seasoned learners—namely, all of us!

## Abbreviations

ICF: International Classification of Functioning, Disability and Health (2001)
PRO: Patient-reported outcomes
PROMS: Patient-reported outcome measures
WHO: World Health Organization
